# Patient medication counselling in community pharmacy: evaluation of the quality and content

**DOI:** 10.1186/s40545-022-00502-3

**Published:** 2022-12-16

**Authors:** Segun Johnson Showande, Monioluwa Wonuola Laniyan

**Affiliations:** Department of Clinical Pharmacy and Pharmacy Administration, Faculty of Pharmacy, University of Ibadan, Ibadan, Nigeria

**Keywords:** Patient medication counselling, Pharmacist, Consumer, United States Pharmacopeia Medication Counselling Behaviour Guideline, Nigeria

## Abstract

**Background:**

Patient medication counselling (PMC) is a pharmaceutical care service targeted at optimizing patient drug use, safety and improving treatment outcomes. This study assessed the content and quality of PMC from the community pharmacists’ (CPs) and pharmacy customers’ (PCs) perspectives.

**Methods:**

A cross-sectional questionnaire-guided survey was conducted in Ibadan, Nigeria, among 125 CPs and 612 PCs. The 35-counselling items validated United States Pharmacopeia Medication Counselling Behaviour Guideline scale with 10-point graded responses (1 = poor to 10 = excellent) was used. Self-reported medication counselling information content provided by CPs and received by PCs was assessed and expressed in median and interquartile ranges. The quality of PMC was evaluated and graded as poor (1–29.9%), unsatisfactory (30–59.9%), satisfactory (60–79.9%) and excellent (80–100%). Associations between demographic variables and overall quality of counselling were determined with Mann–Whitney *U* and Kruskal–Wallis tests at *p* < 0.05.

**Results:**

The response rate was 92.5% and 97.6% for PCs and CPs, respectively. The PCs’ opinions on the individual content of the PMC provided by the CPs were significantly different from the pharmacists’ self-report (*p* < 0.05). Some of the PMC content included how to take the medicine PC = 6.00 (2.00) vs CP = 8.00 (2.00), information on possible side effects PC = 6.00 (2.00) vs CP = 8.00 (2.00), taking history of allergies and other medications PC = 6.00 (6.00) vs CP = 7.00 (1.00), and how to incorporate drug regimen into daily routine PC = 5.00 (6.00) vs CP = 8.00 (3.00). The quality of PMC purportedly provided by CPs and received by the PCs was satisfactory (75%) and unsatisfactory (55%), respectively. The quality of communication counselling offered by CPs trained in Nigeria (Mean rank = 62.49) was higher than those trained outside Nigeria (Mean rank = 26.40), *U* = 228.00, *p* = 0.024. The PC’s age, marital status, and highest educational qualification were significantly associated with their opinion on the quality of counselling received.

**Conclusions:**

Both the community pharmacists and pharmacy customers reported the provision of patient medication counselling on side effects, drug usage, medication history and allergies among others. However, the quality of counselling provided by the pharmacists was satisfactory, but the quality of counselling received by the pharmacy customers was unsatisfactory. Pharmacists may need to engage pharmacy customers more during medication counselling.

## Background

The pharmacy profession has evolved into a more patient-oriented practice involving the provision of care, advice, and medication counselling [1]. Pharmacists are the third largest healthcare professional group in the world [2] and community pharmacists are visited daily by millions of people across the globe. On average, patients visit community pharmacists (CPs) nine times more than they do primary care physicians in a year [3, 4]. They are mostly the first point of contact for some patients and for few others, the only contact point [5]. This provides the CPs with the opportunity to leverage on the interaction with individual patients, intervene in health-related matters, facilitate public health services, and provide other health and well-being services [6]. Both the Omnibus Budget Reconciliation Act of 1990 (OBRA’90) and the code of ethics of the Royal Pharmaceutical Society of Great Britain enjoined pharmacists to be actively involved in patients care by providing information and advice on safe and effective medicine use. [7, 8].

Patient medication counselling (PMC) may be described as “providing medication information orally or in written form to the patients or their representatives or providing proper directions of use, advice on side effects, storage, diet and lifestyle modifications” [9]. The provision of appropriate and adequate PMC by pharmacists could help pharmacists identify and resolve drug therapy problems [10, 11] engage patients in self-management of diseases [12, 13], and prevent treatment failure and limit resource wastage [14, 15]. In developing countries like Nigeria, the provision of such patient-oriented services by pharmacists is still evolving compared to developed nations [16, 17].

The provision of PMC is however compulsory and backed by enabling laws in some countries [18, 19], including monetary penalties for failure to provide PMC for prescription drugs [20]. Yet, pharmacists have often failed to deliver appropriate and detailed medication information to patients [12], despite increased interest in the quality of patient counselling, its propriety, and acceptability in community pharmacies [21]. Though pharmaceutical care is fast becoming the mode of practice in Nigeria, most pharmacists still provide inadequate patient-oriented services including PMC [22]. This may be due to *professional complacency and conservatism, extrinsic system failures, and inadequate human resources for health* as suggested by Abdu-Aguye et al. [23]. There is no PMC policies, guidelines, incentives or standards in the country. However, to enhance the quality of PMC, the Pharmacy Council of Nigeria (PCN) [24], and the National Universities Commission [25] made provision for inclusion of communication skills and patient counselling in the undergraduate curriculum with experiential training at community pharmacies and hospitals during the externship and clerkship programs, respectively. This is also included in one of the modules offered to community and hospital pharmacists in the PCN organized Mandatory Continuing Professional Development [26].

Many professional organizations in the United States of America and Australia have published guidelines on counselling [18, 27, 28] with varying content [29]. Various studies have examined the use of counselling guidelines by pharmacists [30, 31], rate of verbal counselling with author-defined counselling content [32–35], and the content of verbal counselling [36, 37]. Pharmacists behaviors were evaluated in these studies, but others have observed both pharmacists and PCs’ PMC services [32, 38]. As much as it is pertinent to know the number of patients or consumers who received a pre-defined counselling content or medication information, it is equally important to know how comprehensive is the content of the PMC or the quality of the counselling received by the patients or offered by the pharmacists. Most of these cross-sectional studies did not evaluate the quality of pharmacist counselling which considered empathic understanding, acceptance, and the demonstration of genuine feeling during the counselling process [19]. This study, therefore, evaluated the community pharmacists’ and pharmacy customers’ opinions on the content and quality of PMC provided by community pharmacists to PCs using the United States Pharmacopeia Medication Counselling Behaviour Guideline (USP-MCBG) [29].

## Methods

### Study design and setting

This cross-sectional questionnaire-guided survey was carried out in community pharmacies in Ibadan, Nigeria. The city with an estimated population of 3,649,000 as of 2021 is located in the south-western part of the nation in Oyo state [39]. The study lasted for 6 months, from June to November 2021. Participants involved in the study included community pharmacists and customers who visited the pharmacies during the period of the study and who may be patients, caregivers or customers herein referred to as pharmacy customers (PCs).

### Sample size and sampling techniques

A total of 158 pharmacies were registered within Ibadan metropolis, Oyo state, as of December 2020 according to the Pharmacy Council of Nigeria register. Taro Yamane formula was employed to determine the sample size with a 0.05 margin of error and 95% confidence interval with a 10% provision for non-response. The calculated sample size for the community pharmacies was 125.

For the PCs, the sample size was determined by modifying the method described by Showande and Babalola in a study carried out in the same city [1]. Briefly, a conservative estimate of PCs who visited and bought drugs from the selected community pharmacies between 9.00 am and 12.00 pm daily (Monday to Friday) were observed by the authors to be an average of 120. The sample size for each pharmacy was calculated with the 120 PC’s sample frame using Taro Yamane formula to be 95. Furthermore, allowance for a 10% non-response was added and the eventual PCs’ sample size for each community pharmacy was 102. Six community pharmacies with high patronage were purposively selected from six local government area (LGA) out of the 11 LGAs in Ibadan for the PCs’ survey. One community pharmacy per LGA. Four community pharmacies were selected from the five urban LGAs while two were selected from the six semi-urban LGAs in Ibadan. Thus, the total sample size of the PCs surveyed in the six selected community pharmacies was 612.

### Inclusion and exclusion criteria

The survey included the superintendent pharmacists, staff pharmacists, non-intern locum pharmacists and pharmacists undergoing the compulsory 1-year National Youth Service Corps (a year mandatory national program for all graduates less than 30 years old) who gave written informed consent to participate in the study. Intern pharmacists and pharmacy students posted to community pharmacies for externship were excluded from the survey. The PCs, as defined under the study design and setting section, who were 18 years and above, had given verbal informed consent and could read and understand either English or Yoruba language were included in the study. Pharmacy customers who were indisposed to attend to the questionnaire were excluded.

### Study instrument

The PCs’ questionnaire contained sections on respondent characteristics, what the PCs understood about medication counselling and what is expected from pharmacists during medication counselling. It also contained the USP-MCBG scale to evaluate the content and quality of medication counselling provided to PCs by community pharmacists. The USP-MCBG has been used to evaluate patient medication counselling by pharmacists and was found reliable even after modifications [29]. There are 35 items on the scale and the rating of the items utilized 11-point graded responses divided into six categories: 0—not applicable, 1—not done, 2—poor, 3–5—unsatisfactory, 6–7—satisfactory, 8–10—excellent. The “not applicable” grading was not included in the scale used for this study. Thus, the USP-MCBG scale graded responses was from 1 to 10, for this study. The USP-MCBG scale contained four subscales or components; needs assessment, precaution and warnings, management of treatment and communication with 9, 8, 13, and 5 items, respectively.

The same USP-MCBG scale was used for the community pharmacists’ survey but in addition, a 7-item Likert scale with 5 graded responses (strongly disagree—1, strongly agree—5) on the pharmacists’ understanding of the goals of patient medication counselling was included beside the respondent characteristics. Since the pharmacist-participants were literates, translation to a local language was not required. The questionnaire for PCs was translated from English language to Yoruba language and back translated for consistency because some PCs were non-literate in English. The Cronbach alpha reliability coefficient of the scales used in the study ranged from 0.701 to 0.937.

### Study procedure

The pharmacy customers were selected through convenience sampling. A day in a week was picked to conduct the survey in each of the six selected community pharmacies among the pharmacy customers from 9 am to 12 noon. Pharmacy customers exiting the community pharmacy who had purchased a drug were approached, the study was explained to them and consent sort. Those who gave verbal informed consent to participate were given the questionnaire written in English language, but those who understood only Yoruba were assisted by a trained research assistant (a final year pharmacy student) to fill the questionnaire. The questionnaire took about 15 min to fill. If the consent was denied, the next customer coming out of the pharmacy was approached and the sampling continued until the estimated sample size from the pharmacy was reached.

One hundred and twenty-five pharmacies were conveniently selected and one pharmacist per pharmacy who consented to participate was administered the questionnaire which was retrieved after completion on the same day. Where the pharmacist denied verbal consent at the time of visit, another pharmacist on duty was approached or in cases where there was only one pharmacist on duty the next community pharmacy on the list was visited. In another situation where there was no pharmacist on duty on two different visits, the next pharmacy on the list was visited.

But before the main study, the study instruments were reviewed by four pharmacy researchers for relevance, appropriateness, and acceptability. A pre-test was conducted in five pharmacies for all the participants in the study. Based on the comments of the researchers the wordings of the items in the USP-MCBG for the participants were modified to reflect the type of participants being assessed. For example, for item 30 in the USP-MCBG scale, it was captured as “*The pharmacist probed me for additional information*” for PCs, and for community pharmacists, it was written as “*I usually probe for additional information from the patient/customer*”. Five PCs per pharmacy and one pharmacist per community pharmacy were surveyed during the pre-test. No modification was made to the instrument after the pre-test. The data collected during the pre-test were not included in the final analysis.

### Data analysis

Descriptive statistics of sample characteristics and questionnaire items were used to summarize part of the data obtained using mean and standard deviation, median and interquartile range, and proportions with the aid of the Statistical Package for Social Sciences Windows version 26.0 (IBM corp. New York, USA). The quality of counselling was estimated as a percentage: 100 (Mean USP-MCBG scale score or subscale score)/(Total obtainable USP-MCBG scale score or subscale score). For the four subscales or components; needs assessment, precaution and warnings, management of treatment and communication, the total obtainable scores were 90, 80, 130, and 50, respectively. The total obtainable USP-MCBG scale score was 350 (item 34 on the scale has four questions, the average of the scores was considered for item 34). The quality of counselling was further categorized as: poor (1–29.9%), unsatisfactory (30–59.9%), satisfactory (60–79.9%); and excellent (80–100%) based on the graded responses of the USP-MCBG scale. The differences in the USP-MCBG scale content score between the PC’s and the community pharmacist’s self-reports were determined with independent sample *t*-test with consideration for equal variance assumed or not assumed. The association between the socio-demographic variables of the participants and the USP-MCBG subscales and total scores was assessed using Mann–Whitney *U* and Kruskal–Wallis tests. The level of significance was set at *p* < 0.05.

## Results

### Pharmacy customer assessment

Six hundred and twelve questionnaire were distributed and all were retrieved. Forty-six of these questionnaire were unusable due to missing valuable data in the Likert scale. Thus, 566 questionnaire were analyzed. The response rate was therefore 92.5%. The mean age of the PCs was 34.16 ± 13.35 years (range 18–82 years). There was an almost equal distribution of gender with females slightly higher 282 (50.4%). Approximately half, 241 (43.7%), of the PCs had a tertiary education and 116 (21.0%) were employees while 145 (26.3%) were students. Other respondent characteristics are listed in Table 1.

**Table 1:** Demographic characteristics of pharmacy customers

Demographic information	Variables	Frequency (percentage %)	Mean ± SD
Age, years, (*n* = 566)			34.16 ± 13.35
*Age group	≤ 23	192 (33.9)	
	26–44	257 (45.4)	
	45–63	102 (18.0)	
	64+	15 (2.7)	
Gender (*n* = 560)	Male	278 (49.6)	
	Female	282(50.4)	
Religion (*n* = 533)	Christianity	376 (70.5)	
	Islam	155 (29.1)	
	Traditional practice	2 (0.4)	
Marital status (*n* = 557)	Single	283 (50.8)	
	Married	246 (44.2)	
	Widowed	17 (3.1)	
	Divorced	5 (0.9)	
	Separated	1 (1.1)	
Highest academic qualification obtained (*n* = 551)	No formal education	15 (2.7)	
	Primary education	23(4.2)	
	Secondary education	180 (32.7)	
	Tertiary education	241 (43.7)	
	Postgraduate education	92 (16.7)	
Working status (*n* = 552)	Self-employed	132 (23.9)	
	Employee	116 (21.0)	
	Unemployed	27 (4.9)	
	Civil/public servant	41 (7.4)	
	Artisan	18 (3.3)	
	Student	145 (26.3)	
	Farmer	4 (0.7)	
	Trader	28 (5.1)	
	Businessman/woman	37 (6.7)	
	Others	4 (0.7)	

^*^The pharmacy customers’ age was binned based on an equal percentile of scanned cases

Some of the PCs, 194 (34.3%), claim to visit the pharmacies at least once a month. Others visited the pharmacies at least once a week 67 (12.1%), once in 2 weeks 84 (15.1%) while some were visiting the pharmacies for the first time 41 (7.4%). The purpose of the visit included complaining about illness 72 (13.4%), purchasing drugs 372 (69.8%), for medical advice 38 (7.1%), refilling prescriptions 32 (6.0%) and for a scheduled appointment with the pharmacist 10 (1.9%). Most of the PCs, 497 (90.0%), knew that they needed to be counselled on the use of their medications. The PCs, 437 (79.5%), also believed that medication counselling should be provided in community pharmacies. However, few of the PCs, 190 (34.4%), believed that only the pharmacist is in the best place to give information on medications obtained from the pharmacy. The PCs opined that some of the information the pharmacists should provide during medication counselling should include indications 495 (90.0%), what to do with leftover medications 276 (50.5%) and the cost of the medication 386 (70.8%) (see Table 2).

**Table 2:** Pharmacy customers’ understanding of medication counselling

Description	Frequency (%)		
	Yes	No	I don’t know
I am aware that I need to be counselled on the use of medications and other drug/disease-related matters (*n* = 552)	497 (90.0)	39 (7.1)	16 (2.9)
Medication counselling should be provided in community pharmacies (*n* = 550)	437 (79. 5)	49 (8.9)	64 (11.6)
Only the pharmacist is in the best place to give information as regards medications gotten from the pharmacy (*n* = 553)	190 (34.4)	200 (36.4)	163 (29.5)
*I believe that medication counselling involves the pharmacist providing information about*:			
What the medication is supposed to do (550)	495 (90.0)	9 (1.6)	46 (8.4)
How long the medication is to be taken (*n* = 549)	486 (88.5)	15 (2.7)	48 (8.7)
How to properly store the medication (*n* = 550)	389 (70.7)	83 (15.1)	78 (14.2)
The way the medication was manufactured (*n* = 547)	120 (21.9)	287 (52.5)	140 (25.6)
The company that produced the medication (*n* = 546)	135 (24.7)	266 (48.7)	145 (26.6)
What to do with leftover medications (*n* = 546)	276 (50.5)	163 (29.9)	107 (19.6)
Proper diet and how to adjust my lifestyle (*n* = 547)	415 (75.9)	66 (12.1)	66 (12.1)
The cost of the medication (*n* = 555)	386 (70.8)	83 (15.2)	76 (13.9)

### Pharmacist self-evaluation

One hundred and twenty-five questionnaire were distributed and 122 were retrieved and analyzed. Three pharmacists were unable to complete and submit the questionnaire. The response rate was 97.6%. The average age of the pharmacists was 34.54 ± 12.07 years (range 23–85 years), 76 (63.3%) were males, and only 5 (4.1%) were trained outside Nigeria (Table 3). The mean year of community pharmacy experience was 6.31 ± 8.69 years. The community pharmacists agreed that patient counselling should comprise both written and oral provision of medication information to PCs 113 (92.6%) and it should be a one-on-one interaction 112 (91.8%). Pharmacists opined that medication counselling should provide information on the appropriate use of medication 115 (94.3%), it should entail being involved in the patient’s social, dietary, psychological and emotional needs 102 (83.6%) and good communication is a vital tool to achieve optimal patient medication counselling 115 (94.3%). Community pharmacists are stakeholders concerning medication and medication-related information 116 (95.1%).

**Table 3:** Demographic characteristics of community pharmacists

Demographic information	Variables	Frequency (%)	Mean ± SD
Age, years			34.54 ± 12.07
*Age group (*n* = 95)	≤ 25	13 (13.7)	
	26–45	68 (71.6)	
	46–65	11 (11.6)	
	66+	3 (3.2)	
Gender (*n* = 120)	Male	76 (63.3)	
	Female	44 (36.7)	
Country of pharmaceutical training (*n* = 121)	Nigeria	116 (95.9)	
	Outside Nigeria	5 (4.1)	
Cadre of pharmacist (*n* = 118)	Superintendent pharmacist	52 (44.1)	
	NYSC pharmacist	7 (5.9)	
	Post-intern locum pharmacist	9 (7.6)	
	Pharmacy manager	21 (17.8)	
	Staff pharmacist	29 (24.6)	
Highest educational qualification (*n* = 122)	B.Pharm	94 (77.0)	
	Pharm. D	9 (7.4)	
	M.Pharm/M.Sc	13 (10.7)	
	FPCPharm	3 (2.5)	
	MBA	1 (0.8)	
	Ph.D	2 (1.6)	
Practiced in other pharmacy setting (*n* = 120)	Yes	99 (82.5)	
	No	21 (17.5)	
Previous area of practice (*n* = 102)	Research/Academia	4 (3.9)	
	Hospital	69 (67.6)	
	Government and NGOs	3 (2.9)	
	Pharmaceutical industry	12 (11.8)	
	Sales representative	13 (12.7)	
	Public health	1 (1.0)	
*Years of post-NYSC community practice Group (*n* = 108)			6.33 ± 9.34
	≤ 5	74 (68.5)	
	6–17	24 (22.2)	
	18–29	1 (0.9)	
	30+	9 (8.3)	
*Years of community pharmacy experience Group (*n* = 121)			6.31 ± 8.69
	≤ 5	84 (69.4)	
	6–20	29 (24.0)	
	21–35	5 (4.1)	
	36+	3 (2.5)	
*Estimated number of prescriptions filled or seen in the pharmacy per day (*n* = 113)			36.80 ± 48.93
	≤ 50	94 (83.2)	
	51–140	16 (14.2)	
	141–230	1 (0.9)	
	231+	2 (1.8)	
*Estimated number of PCs attended to per day (*n* = 116)			85.16 ± 129.01
	≤ 100	94 (81.0)	
	101–400	20 (17.2)	
	701+	2 (1.7)	
*Estimated time spent with each pharmacy customer (min) Group (*n* = 120)			8.38 ± 4.87
	≤ 2	5 (4.2)	
	3–8	59 (49.2)	
	9–14	37 (30.8)	
	15+	19 (15.8)	

*MBA* Master in Business Administration, *PCs* pharmacy customers, *B.Pharm* Bachelor of Pharmacy, *PharmD* Doctor of Pharmacy, *FPCPharm* Fellow Postgraduate College of Pharmacists, *M.Sc* Master of Science, *NYSC* National Youth Service Corps, *NGO* Non-Governmental Organization, *M.Pharm* Master of Pharmacy

^*^Binned variables based on equal width interval of scanned cases

### Pharmacists’ and pharmacy customers’ opinions on the content and quality of patient medication counselling

The PCs’ opinions on the individual content of the PMC provided by community pharmacists were significantly different from the pharmacists’ self-report (*p* < 0.05); see Table 4. In terms of needs assessment, the community pharmacists claim to ask the PCs for the history of allergies or other medications 7.00 (1.00)–[Median (interquartile range)], respond with empathy to PC’s concerns 8.00 (3.00), and ask about medical, family and social history 7.00 (1.00). However, the PCs claimed that the provision of this counselling was satisfactory, but the use of appropriate counselling aids to support medication counselling by the community pharmacists was poor 2.00 (5.00).

**Table 4:** Pharmacists’ and pharmacy customers’ opinions on the content of pharmacist’s patient medication counselling

Patient medication counselling content (USP-MCBG)	Median (IQR)	
	Pharmacy customers *N* = 566	Pharmacists *N* = 122
Component 1: Needs assessment		
The pharmacist asked me if I had allergies, any other medications taken, my age, etc	6.00 (6.00)	7.00 (1.00)
The pharmacist responded with understanding/empathy to my questions and concerns	6.00 (5.00)	8.00 (3.00)
The pharmacist asked questions about my illness, family and social history prior to counselling	6.00 (7.00)	7.00 (1.00)
The pharmacist explained the purpose of the counselling session to me	6.00 (6.00)	7.00 (2.00)
The pharmacist presented facts and concepts concerning the drug and my illness in a logical order	6.00 (6.00)	8.00 (2.00)
The pharmacist used appropriate counselling aids such as pictures to support the counselling	2.00 (5.00)	7.00 (2.00)
The pharmacist assessed any actual and/or potential concerns or problems of importance	6.00 (6.00)	7.00 (3.00)
The pharmacist determined if I have any other medical conditions which could influence the effects of this drug or influence the likelihood of an adverse reaction	6.00 (3.00)	8.00 (2.00)
The pharmacist conducted appropriate counselling introduction by identifying him/herself and me	6.00 (6.00)	7.00 (2.00)
Component 2: Precautions and warnings		
The pharmacist explored with me potential problems in taking the medication as prescribed (e.g., cost, access, etc.)	6.00 (2.00)	8.00 (3.00)
The pharmacist discussed potential (significant) side effects	6.00 (2.00)	8.00 (2.00)
The pharmacist warned me or the patient concerned about taking other medications, including OTCs, herbals/botanicals and alcohol, which could inhibit or interact with the prescribed medication	6.00 (2.00)	8.00 (2.00)
The pharmacist discussed significant interactions that might occur when using my drugs with certain drugs, food and other diseases	7.00 (1.00)	8.00 (3.00)
The pharmacist discussed precautions to take (activities to avoid, etc.)	6.00 (2.00)	8.00 (2.00)
The pharmacist explained in precise terms what to do if the patient misses a dose	6.00 (6.00)	7.00 (2.00)
The pharmacist discussed how to prevent or manage the side effects of the drug if they do occur	6.00 (6.00)	7.00 (2.00)
The pharmacist helped me to generate solutions to potential problems	6.00 (6.00)	7.00 (2.00)
Component 3: Management of the treatment		
The pharmacist discussed the storage recommendations, ancillary instructions with respect to the medications (e.g., shake well, refrigerate, etc.)	6.00 (4.00)	8.00 (3.00)
The pharmacist explained how long it will take for the drug to show an effect	6.00 (4.00)	7.00 (2.00)
The pharmacist told me when I am due for a refill/repurchasing of my medications	6.00 (7.00)	8.00 (3.00)
The pharmacist summarized the session by acknowledging and/or emphasizing key points of information	6.00 (6.00)	8.00 (2.00)
The pharmacist emphasized the benefits of completing the medication as prescribed	7.00 (3.00)	9.00 (2.00)
The pharmacist helped me to plan follow-up and next steps	6.00 (5.00)	8.00 (3.00)
The pharmacist provided an opportunity for final concerns or questions	6.00 (6.00)	8.00 (2.00)
The pharmacist verified if I understood by requesting feedback or a recap	6.00 (6.00)	8.00 (3.00)
The pharmacist maintained control and direction of the counselling session	6.00 (4.00)	8.00 (3.00)
The pharmacist assisted me in developing a plan to incorporate the medication regimen into my daily routine	5.00 (6.00)	8.00 (3.00)
The pharmacist used open-ended questions that required me giving explanation	6.00 (6.00)	8.00 (3.00)
The pharmacist explained to me the way I will take my medications including scheduling and duration of the medication use when appropriate	6.00 (2.00)	8.00 (2.00)
The pharmacist probed me for additional information	6.00 (6.00)	8.00 (2.00)
Component 4: Communication		
The pharmacist communicated in a language that I understood	7.00 (2.00)	9.00 (2.00)
The pharmacist provided me with accurate information	6.00 (2.00)	9.00 (2.00)
The pharmacist discussed with me the name of the medication and what I’m using it for	7.00 (2.00)	9.00 (3.00)
The pharmacist displayed effective nonverbal behaviors	7.00 (2.75)	8.38 (2.00)
The pharmacist assessed my understanding of the reason(s) for the therapy	6.00 (8.00)	8.00 (2.00)

1—not done, 2—poor, 3–5—unsatisfactory, 6–7—satisfactory, 8–10—excellent

*USP-MCBG* United States Pharmacopeia Medication Counselling Behaviour Guideline, *IQR* interquartile range

As shown in Table 4, the community pharmacists also claim to provide excellent counselling on precautions and warnings such as discussion on potential side effects of drugs with PCs 8.00 (2.00) and warning about taking other medications or herbs with the drug 8.00 (2.00). Other satisfactory counsellings provided were what to do with missed doses 7.00 (2.00) and how to prevent or manage side effects 7.00 (2.00). The PCs were also satisfied with the provision of these counselling. In terms of the provision of counselling on the management of treatment, the PCs were not satisfied that the pharmacists did not factor their drug regimen into their daily routine 5.00 (6.00), however, they were satisfied with the counselling that the pharmacists provided on when the effect of the drug will be seen 6.00 (4.00) and how to take the medications 6.00 (2.00). The community pharmacists nevertheless claimed that all the content of the counselling under the management of the treatment was provided to the PCs in detail. Both community pharmacists and PCs agreed that the pharmacists provided satisfactory medication counselling by communicating in understandable language and with nonverbal behaviors. The opinions of the PCs and the community pharmacists on other contents of the medication counselling provided by the community pharmacists are outlined in Table 4.

Relating to the quality of PMC provided by the community pharmacists, Table 5 shows that the PCs were not satisfied with the overall counselling. The mean percent quality counselling for the entire USP-MCBG scale was 54.78 ± 18.44% compared with the pharmacist 75.00 ± 12.41% which was satisfactory (*p* < 0.001). In terms of the communication component of the USP-MCBG scale, the PCs were satisfied with the quality of counselling provided under this section 66.20 ± 15.62%.

**Table 5:** Differences in the perceived quality of counselling provided by pharmacists and received by pharmacy customers

USP-MCBG scale	Description	Mean ± SD		*p*-value*
		Pharmacist *N* = 122	Pharmacy customers *N* = 566	
Component 1	Needs assessment score	64.05 ± 10.76	43.82 ± 19.73	< 0.001
	Mean quality of counselling (%)	71.17 ± 11.96	48.68 ± 21.92	< 0.001
	Grading	Satisfactory	Unsatisfactory	
Component 2	Precautions and warnings	58.98 ± 11.63	45.61 ± 15.41	< 0.001
	Mean quality of counselling (%)	73.73 ± 14.54	57.02 ± 19.26	< 0.001
	Grading	Satisfactory	Unsatisfactory	
Component 3	Management of the treatment	98.00 ± 20.21	69.22 ± 28.12	< 0.001
	Mean quality of counselling (%)	75.39 ± 15.54	53.24 ± 21.63	< 0.001
	Grading	Satisfactory	Unsatisfactory	
Component 4	Communication	41.47 ± 7.25	33.10 ± 7.81	< 0.001
	Mean quality of counselling (%)	82.93 ± 14.50	66.20 ± 15.62	< 0.001
	Grading	Excellent	Satisfactory	
USP-MCBG	Total score	262.50 ± 43.45	191.74 ± 64.53	< 0.001
	Mean quality of counselling (%)	75.00 ± 12.41	54.78 ± 18.44	< 0.001
	Grading	Satisfactory	Unsatisfactory	

Quality of counselling grading: poor (1–29.9%), unsatisfactory (30–59.9%), satisfactory (60–79.9%); excellent (80–100%)

*USP-MCBG* United States Pharmacopeia Medication Counselling Behaviour Guideline

^*^Independent sample *t*-test, *p* < 0.05 is considered significant

A Mann–Whitney *U* test revealed a significant difference in the perceived quality of needs assessment counselling provided by male pharmacist (mean rank = 68.67, *n* = 76) and female pharmacist (mean rank = 46.39, *n* = 44), *U* = 1051, *p* = 0.001). Also, the quality of communication counselling provided to PCs by community pharmacists differ based on where the pharmacists were trained. The quality of counselling offered by community pharmacists trained in Nigeria (mean rank = 62.49, *n* = 116) was higher than those trained outside Nigeria (mean rank = 26.40, *n* = 5), *U* = 228.00, *p* = 0.024 (Table 6). For the PCs, Table 7 shows that PC’s age, marital status, highest educational qualification and working status were significantly associated with the opinion of PCs on the quality of counselling provided by the community pharmacists (*p* < 0.05).

**Table 6:** Association between pharmacists’ demographic variables and perception of the quality of counselling provided

Demographics	Categories	*N*	Quality of patient medication counselling based on the USP-MCBG scale Mean ranks				
			Component 1	Component 2	Component 3	Component 4	USP-MCBG total score
Gender	Male	76	68.67	63.18	62.72	59.91	64.13
	Female	44	46.39	55.88	56.66	61.51	54.23
	*p*-value^a^		0.001*	0.267	0.357	0.808	0.133
Age, years	≤ 25	13	44.08	50.88	51.69	58.65	51.73
	26–45	68	47.40	47.38	47.84	46.13	47.38
	46–65	11	55.45	49.05	46.14	47.18	47.77
	66+	3	51.17	45.67	42.50	47.17	46.67
	*p*-value^b^		0.767	0.975	0.939	0.519	0.964
Country of pharmacy training	Nigeria	116	61.53	62.24	62.09	62.49	62.19
	Outside Nigeria	5	48.60	32.30	35.60	26.40	33.40
	*p*-value^a^		0.419	0.062	0.098	0.024*	0.072
Cadre of pharmacists	Superintendent pharmacist	52	54.05	54.62	55.83	55.19	54.40
	NYSC pharmacist	7	70.50	64.64	61.79	70.57	66.71
	Post internship locum pharmacist	9	54.33	65.39	62.33	62.22	60.11
	Pharmacy manager	21	74.71	68.33	64.67	63.83	68.90
	Staff pharmacist	29	57.21	58.79	60.91	60.57	59.90
	*p*-value^b^		0.162	0.574	0.874	0.739	0.548
Years of community pharmacy experience	≤ 5	84	60.43	62.13	61.26	63.70	61.98
	6–20	29	63.26	61.90	64.97	58.47	62.97
	21–35	5	61.40	52.60	45.80	48.20	46.70
	36+	3	54.50	34.83	40.67	31.17	38.33
	*p*-value^b^		0.970	0.559	0.508	0.322	0.524
Estimated time spent with PCs (min)	≤ 2	5	60.80	88.20	90.20	63.90	81.10
	3–8	59	56.86	61.97	61.08	62.85	60.80
	9–14	37	67.55	57.22	59.24	59.27	60.26
	15+	19	58.00	55.03	53.34	54.71	54.63
	*p*-value^b^		0.518	0.253	0.211	0.828	0.512

*USP-MCBG* United States Pharmacopeia Medication Counselling Behaviour Guideline

^a^Mann–Whitney *U* test

^b^Kruskal–Wallis test

**Table 7:** Association between pharmacy customers’ demographic variables and perception of the quality of counselling received

Demographics	Categories	*N*	Quality of patient medication counselling based on the USP-MCBG scale Mean ranks				
			Component 1	Component 2	Component 3	Component 4	USP-MCBG total score
Gender	Male	278	273.74	277.53	269.89	271.10	271.12
	Female	281	286.19	282.45	290.01	288.80	288.79
	*p*-value^a^		0.362	0.719	0.141	0.195	0.196
Age, years	≤ 25	192	400.08	405.30	415.02	360.72	417.13
	26–44	257	269.04	263.68	263.00	280.17	262.68
	45–63	102	133.30	133.62	120.49	150.35	117.40
	64+	15	60.33	83.10	59.83	257.57	59.23
	*p*-value^b^		< 0.0001	< 0.0001	< 0.0001	< 0.0001	< 0.0001
Marital status	Single	283	355.36	366.09	369.24	334.06	370.25
	Married	246	208.87	195.13	191.98	226.45	191.35
	Widowed	17	99.59	115.88	99.62	179.24	98.41
	Divorced	5	147.90	150.10	165.20	219.30	152.10
	Separated	6	170.50	179.42	193.67	169.33	186.17
	*p*-value^b^		< 0.0001	< 0.0001	< 0.0001	< 0.0001	< 0.0001
Highest academic qualification	No formal education	15	232.60	174.43	219.17	249.43	207.47
	Primary education	23	234.13	246.00	261.70	273.98	249.93
	Secondary education	180	345.66	342.88	345.15	316.92	350.43
	Tertiary education	241	255.51	260.40	253.97	259.53	253.70
	Post graduate education	92	210.92	210.06	211.25	243.91	206.49
	*p*-value^b^		< 0.0001	< 0.0001	< 0.0001	< 0.0001	< 0.0001
Working status	Self-employed	132	204.81	212.12	207.69	227.31	204.51
	Employee	116	227.10	225.75	215.63	233.75	220.14
	Unemployed	27	250.35	271.87	251.04	291.48	258.67
	Civil/public servant	41	189.18	168.96	179.84	231.43	174.84
	Artisan	18	220.75	199.94	223.75	210.83	209.22
	Student	145	368.17	370.22	380.93	318.03	382.16
	Farmer	4	286.63	156.88	244.25	199.63	236.13
	Trader	28	157.75	159.04	142.75	201.04	143.16
	*p*-value^b^		< 0.0001	< 0.0001	< 0.0001	< 0.0001	< 0.0001

*USP-MCBG* United States Pharmacopeia Medication Counselling Behaviour Guideline

^a^Mann–Whitney *U* test

^b^Kruskal–Wallis test

## Discussion

Assessing the content and quality of PMC using an established guideline shows the comprehensiveness of the medication information given to PCs. This is supported by the study among Finnish community pharmacists where 70% of the pharmacists considered the USP-MCBG useful in PMC [29]. Our study evaluated the opinion of the PMC provider (the community pharmacists, CPs) and the receiver (the pharmacy consumers, PCs) on the content of medication counselling and subsequently determined the quality of the medication information given or received. To the best of our knowledge, this is the first study in Nigeria focusing on this perspective of PMC.

The findings in this study showed that both the provider and the recipient of PMC agreed that the community pharmacist asked about the patient’s allergies, medical, family, and social history. In Saudi Arabia, pharmacists showed little or no concern about taking the history of drug allergy [40]. However, in this same study and another in Ethiopia, pharmacists claimed to take medication history during PMC which is similar to our report, but contradicts the report from another study in Nigeria by Osemene et al. [41] where most of the pharmacists did not take patients medication history. Pharmacists can easily identify the error in patients’ self-reported medication history. But when inaccurate and inadequate information on patient medication history and allergies is gathered during PMC, this may hamper the quality of patient care, cause medication errors and affect patient safety. Pharmacists may be unable to promptly prevent inappropriate drug therapy, drug interactions and possible treatment duplications [42].

There was mutual satisfaction between the community pharmacists and PCs on the provision of PMC on side effects, drug interactions and what to do with leftover medications. There are different reports on the provision of information on medication side effects to patients or consumers and by pharmacists. Some consumers prefer to receive the information on side effects [43–45], some pharmacists prefer not to give it at all [19, 46] while some others seldom provide the information on side effects to consumers [14, 47]. These different reports may be due to the type of population studied, the content of the medication counselling, the use of medication counselling guidelines, the practice setting and whether the medication counselling was for prescription or over-the-counter drugs. Notwithstanding, counselling patients on the side effects of medication is pertinent. Consumers’ or PCs’ knowledge of expected side effects (s) may help them to promptly identify and report such side effects to the pharmacists or other healthcare professionals. This may impel the pharmacist to identify and report adverse drug reactions and identify and resolve drug therapy-related problems. Thus, pharmacists should be able to pass appropriate and balanced information on the beneficial effects and side effects of medications to PCs [48].

Contrary to the report in a study in Vermont USA where 76% of the participants were not informed by the pharmacist on what to do with leftover medications [49], the PCs agree with the pharmacists that this information was satisfactorily provided. This is important as improperly disposed pharmaceuticals have been reported to have untold consequences on the environment [49, 50]. Patients or PCs equipped with the knowledge of the impact that drugs disposed of improperly could have on the environment are more likely to return leftover medications for proper disposal. Thus, it is paramount that a protocol such as a drug-take-back program is established for the proper disposal of unused and leftover medications in Nigeria and around the world [50].

The PCs however agreed with the pharmacists on the provision of counselling on how to take the medication and when the effect of the drug will be seen. In most studies on PMC, information on the direction for use, dose, name of medicine, and indications, were frequently given [6, 14, 51]. This is probably what most community pharmacists considered the minimum PMC information required by the patient or consumer. This is essential for the rational use of medicines.

Patients or consumers have always been viewed to play a passive role of help seeker in patient medication counselling [52–54] and are seldom encouraged to ask questions during counselling [55, 56]. This tallies with the claim by the PCs that the community pharmacists did not give them enough opportunity to express their concerns or ask questions. Studies showed that patients are interested in receiving personalized information on the use of their medication but their intention to initiate consultation or ask questions during counselling is suboptimal [57–59]. Nevertheless, motivating patients’ or consumers’ participation in the counselling process assists in identifying drug therapy-related problems, improving patients’ knowledge of disease and medication, and enhancing patient understanding [10].

Summarily the quality of counselling provided by the pharmacists was satisfactory, but the quality of counselling received by the PCs was unsatisfactory. This finding is similar to that conducted in South Korea by Yang et al. [12]. As reported by Yang et al., the major reason why PCs were not satisfied with the PCs’ PMC was insufficient time for counselling [12]. Several other reasons have been adduced in the literature and these include PCs forgetting the information provided by the pharmacist, lack of interest or refusing counselling [60, 61], type of PC (new or old) as new PCs are likely to receive more PMC than old PCs [62], and prescription (new or refill) since pharmacy customers with new prescription are likely to be counselled more than those with refill prescription because the pharmacist may assume that the PC on chronic medication(s) has detailed information about the medication(s) [19, 60, 62, 63].

Contrariwise, pharmacists gave a higher perceived quality of counselling possibly due to over-reporting of their counselling services to fulfill social expectations [64]. But pharmacists tend to provide more PMC to PCs than other pharmacy staff because of their knowledge on drug therapy [60, 63, 65]. Factors that affect the quality of counselling are the type of prescription (new or refill), pharmacy busyness, pharmacists perceived patients need [66], and the type of consultation with the pharmacist (product request, symptom-based consultation, prescription counselling, etc.), others include lack of privacy [67], lack of or inadequate remuneration [60], patient relying on the physician recommendation more than the pharmacists counselling [67], patient factors (such as the nature of illness and interest to receive counselling) [60], and time constraint [68]. Pharmacists tend to pay attention to drug more than patients, and pharmacists may assume that patients have prior knowledge on the drug and may feel that the provision of additional information may be construed as patronizing and thus the pharmacists may fear negative patient reaction. All these affect the content and quality of PMC provided by pharmacists.

Most studies described the rate of counselling rather than the quality of counselling as reported in this study. The rate of counselling was reported as the proportion of consumers who received verbal counselling and ranged from 8 to 56% [7, 35, 38] for consumers and 51 to 100% [69–71] for pharmacists. In comparison, the quality of counselling received by the PCs was 55% and that provided by the community pharmacists was 75%. Though the figures may not be comparable because of the differences in the definitions of the rate of counselling and quality of counselling, there seems to be a similar trend. For the rate of counselling, pharmacist > consumer, and also for the quality of counselling, pharmacist > pharmacy customers. The type of information considered for the provision of counselling varies across studies. Some studies considered more than 5 items, others focused on 3 items, some studies investigated the type of information given to consumers [14, 34, 36]. But in this study, 35 items were considered based on the USP-MCBG scale. This affords a comprehensive coverage of PMC.

It is obvious from this study that there is the disparity in the quality of counselling purportedly provided by the community pharmacists and that received by PCs. Though from the literature the difference is expected because of the self-report nature of the study. Improvement in the quality of counselling may be enhanced with education, patient-centered communication [72, 73], evaluation of pharmacy practice with feedbacks [62] and the promotion and adoption of the use of counselling guidelines like USP-MCBG. Presently there is no specific patient PMC guideline used by pharmacists in the country. Thus, there is a need for consensus counselling guidelines.

## Strength and limitations of the study

The strength of the study lies in the use of a validated medication counselling guideline to determine the content and quality of PMC offered by community pharmacists and received by PCs. This study did not consider written medication counselling in the evaluation of the content and quality of counselling, thus the report may be lower compared with when both written and verbal medication counselling is considered. Likewise, the provision and receipt of medication counselling content were based on pharmacists’ and PCs’ self-reports, respectively. This is prone to social desirability and recall biases. Another limitation of the study is the employment of a convenient sampling method which may introduce selection bias especially in the PCs’ survey where they were sampled only for a day in each pharmacy. The full remit of PCs within the study period was not feasible with this sampling. Also, the study was carried out among community pharmacists and pharmacy customers in a city in one of the southwest states in Nigeria, thus generalization of the result may be limited. The findings presented in this study should thus be considered in light of these limitations.

## Conclusion

Both the community pharmacists and the pharmacy customers agreed that information on drug usage, side effects, drug interactions, and what to do with leftover medication(s) was provided during patient medication counselling. Overall, the quality of counselling provided by the community pharmacists was satisfactory, but the quality of counselling received by the pharmacy customers was unsatisfactory.

## Data Availability

The datasets used or analyzed during the current study are available from the corresponding author on reasonable request.

## Abbreviations

PMC: Patient medication counselling
PC: Pharmacy customer
CP: Clinical pharmacist
USP-MCBG: United States Pharmacopeia Medication Counselling Behaviour Guideline
